# Rice Body Formation Within a Peri-Articular Shoulder Mass

**DOI:** 10.7759/cureus.718

**Published:** 2016-08-01

**Authors:** Michele N Edison, Anthony Caram, Miguel Flores, Kurt Scherer

**Affiliations:** 1 Diagnostic Radiology, Florida Hospital-Orlando; 2 College of Medicine, UCF College of Medicine

**Keywords:** rice bodies, rheumatoid arthritis, bursitis, magnetic resonance imaging, ultrasound

## Abstract

Most commonly associated with chronic inflammatory conditions, rice bodies represent an uncommon, nonspecific, often intra-articular inflammatory process. Presumably, rice bodies represent the sequelae of microvascular infarcts of the joint synovium. However, rice bodies have been seen in pleural fluid, in the setting of bursitis, and within the tendon sheath. The etiology and prognostic significance of rice bodies are not clear. MRI is the diagnostic imaging modality of choice for the evaluation of rice body formation. Here we present a case of a 28-year-old female with a history of rheumatoid arthritis (RA) who presented to her primary care physician with a palpable mass around her right shoulder which was presumed to be a lipoma. An initial ultrasound showed a fluid filled structure with internal debris. Subsequent MRI evaluation was confirmatory for subacromial-subdeltoid bursitis with rice body formation. The salient point of this report is to highlight the importance of patient-specific differential diagnosis. While lipomas are a very common benign soft tissue tumor, patients with RA often have disease-specific sequelae that should be included in the diagnostic deliberation. Thus, when ordering diagnostic testing for patients with a palpable mass and rheumatoid arthritis, MRI--possibly preceded by conventional radiography--is the most appropriate diagnostic algorithm.

## Introduction

Rice body formation represents an uncommon, nonspecific, inflammatory process [1-3]. Formation of rice bodies is most commonly associated with rheumatoid arthritis (RA), tuberculosis (TB), juvenile arthritides, seronegative arthritis, osteoarthritis, septic joint, trauma, and chronic bursitis [1-2]. Rice bodies were initially identified in 1895 in a patient with TB infection [1-2]. Grossly, rice bodies appear as grains of polished rice [1-3]. They are composed of an acidophilic collagenous center and are encased in fibrin [1]. Rice bodies are presumed to represent the sequelae of microvascular infarcts of the joint synovium, which after sloughing off become encased in layers of fibrin [1, 4]. However, rice body formation has been seen in pleural fluid, within bursae, and in association with the tendon sheath [1]. Because rice bodies have been found in multiple extra-articular locations, some believe that activation of fibroblasts leads to collagen formation which subsequently becomes encased in fibrin [1]. Others believe that the collagenous component arises from bursal tissue [2]. The exact etiology and prognostic significance of rice body formation are still uncertain as the disease severity and longevity do not seem to be correlated [1].

## Case presentation

**Figure 1:**
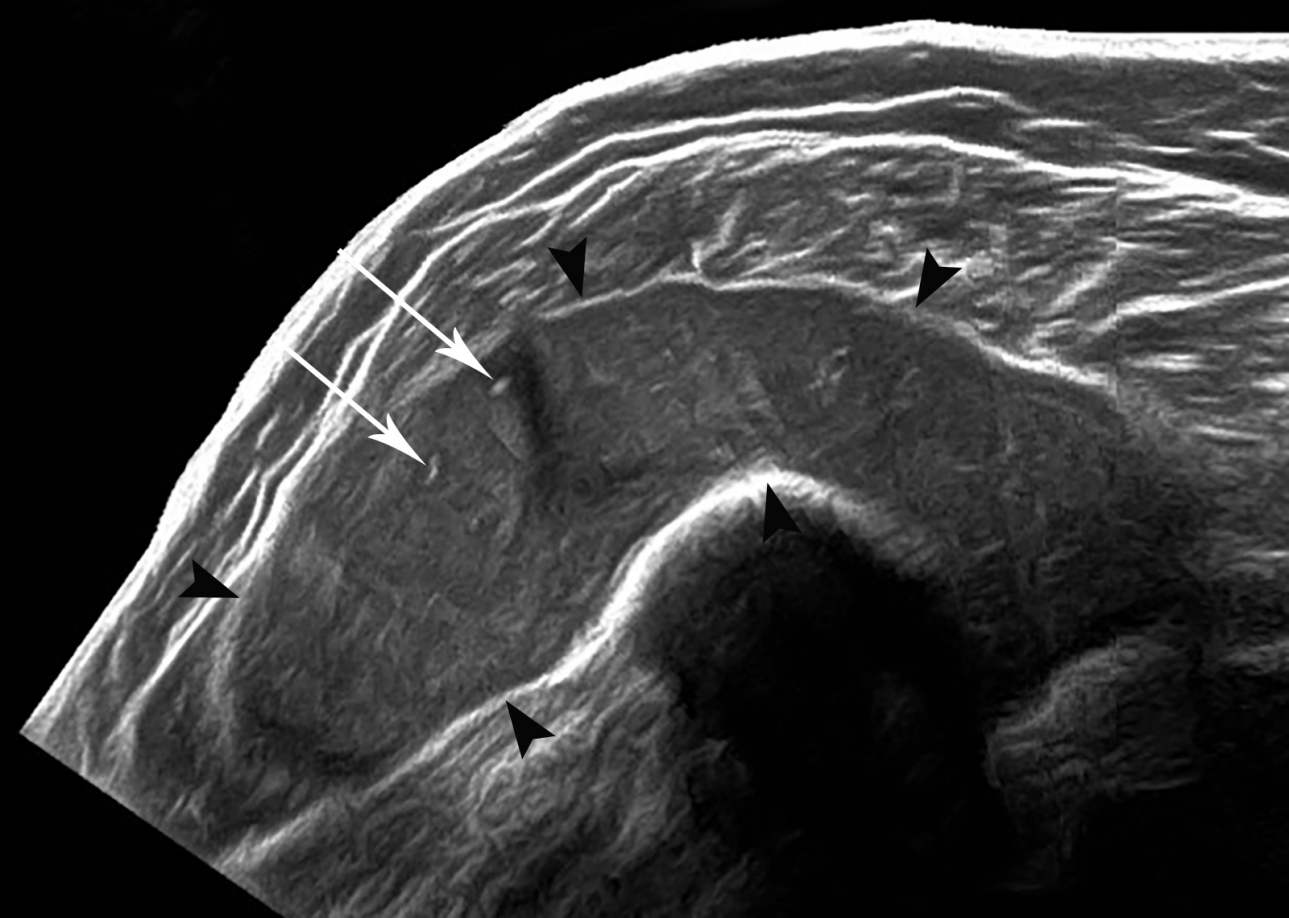
Grayscale Ultrasound (US) Imaging of the Right Shoulder in a 28-Year-Old Female with Shoulder Mass. US demonstrates a well-circumscribed, mildly hypoechoic 7.8 cm mass abutting the deltoid muscle (black arrowheads) with multiple internal hyperechoic 'flecks' (white arrows).

A 28-year-old Caucasian female with a history of RA presented to her primary care physician with a palpable mass around her right shoulder. Her physician presumed the mass to be a lipoma and subsequently ordered a right shoulder ultrasound. Ultrasound imaging showed a well-circumscribed, mildly hypoechoic, 7.8 cm mass abutting the deltoid muscle (Figure 1).

**Figure 2:**
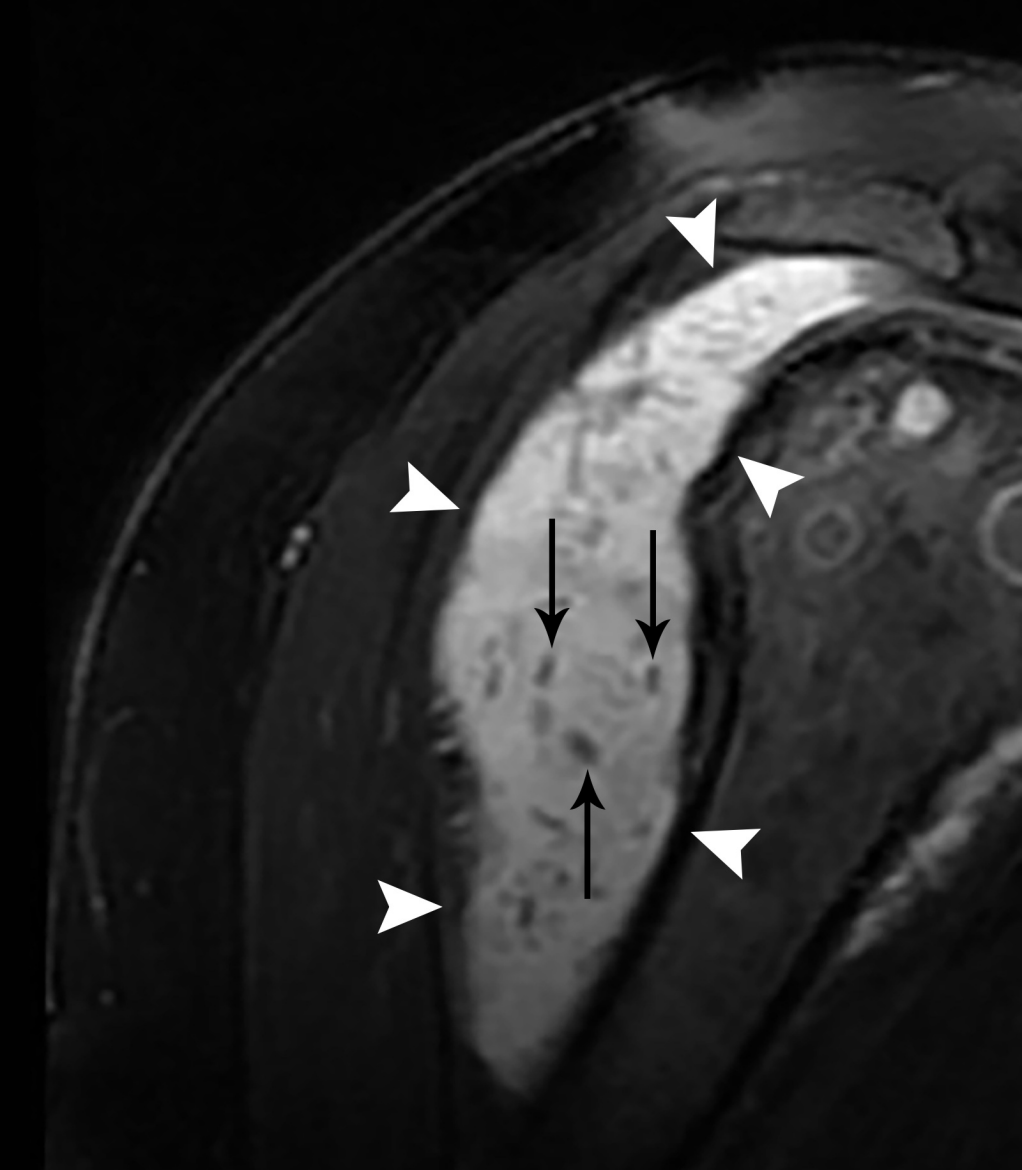
Magnetic Resonance Imaging (MRI) Imaging of a Shoulder Mass in a 28-Year-Old Female. A T2 fat-saturated sequence demonstrates a 10.6 cm hyperintense fluid collection (white arrowheads) with multiple foci of internal debris, consistent with rice body formation (black arrows).

There were multiple internal hyperechoic 'flecks' noted within this mildly hypoechoic mass. The initial imaging characteristics were nonspecific—not classic for a lipoma, but also not entirely exclusive. Also, a malignant etiology would not be ruled out by ultrasound alone. Thus, cross-sectional imaging was recommended to evaluate this solid-appearing, right shoulder mass further. The subsequent magnetic resonance imaging (MRI) showed a 10.6 cm, circumscribed, T2-weighted imaging hyperintense collection surrounding the shoulder joint with multiple hypointense internal flecks (Figure 2).

**Figure 3:**
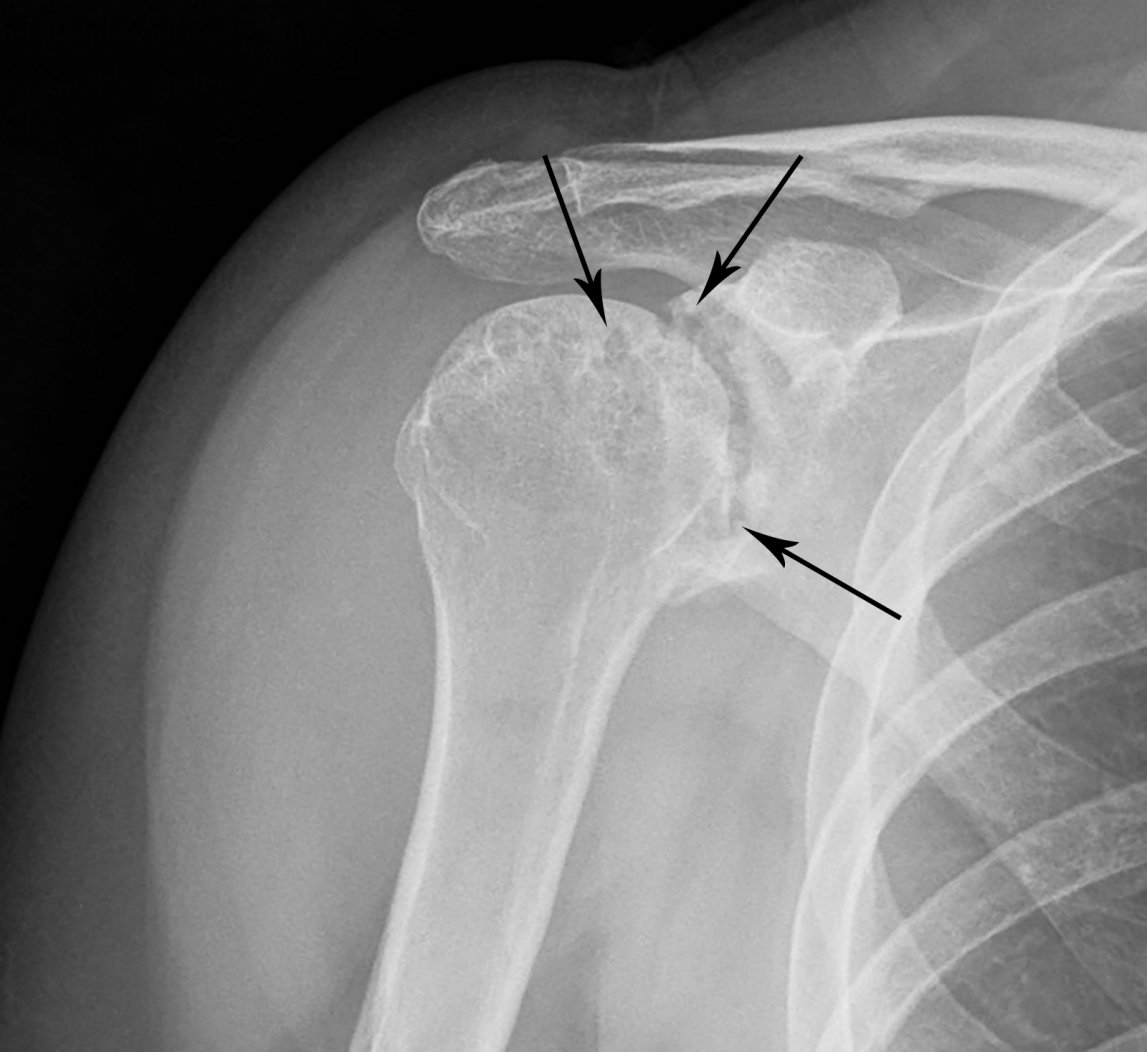
Radiograph of the Right Shoulder in this 28-Year-Old Female with Rheumatoid Arthritis. There are numerous periarticular joint erosions (arrows) and joint space narrowing which is consistent with diagnosis of with rheumatoid arthritis.

This appearance is consistent with a fluid collection with foci of internal debris most consistent with a diagnosis of rheumatoid-arthritis-associated subacromial-subdeltoid bursitis with rice body formation. Subsequently, a conventional radiograph of the right shoulder was obtained to evaluate the osseous structures, which demonstrated marked narrowing of the glenohumeral joint as well as a significant burden of erosions (Figure 3).

The patient agreed to participate and was explained the nature and objectives of this study, and informed consent was formally obtained. No reference to the patient's identity was made at any stage during data analysis or in the report.

## Discussion

Regarding diagnostic imaging, MRI is the modality of choice for evaluation of rice body formation [1]. By appearance on imaging, two reasonably common joint pathologies--pigmented villonodular synovitis (PVNS) and synovial chondromatosis--should be included in the differential diagnosis of intra-articular rice bodies [1-2]. Rice bodies are low to intermediate signal on T1-weighted sequences and low signal on T2-weighted sequences [1-2]. Synovial chondromatosis, however, is hyperintense on T2-weighted imaging [2]. PVNS can be differentiated using gradient echo sequences [1]. Due to the presence of hemosiderin within PVNS lesions, there is susceptibility artifact on gradient echo sequences in patients with PVNS, but not in patients with rice bodies [1].

As stated previously, rice body formation is associated with multiple inflammatory conditions including chronic arthritides, infection, and even trauma [1-2]. However, a few patients have presented with rice body formation before development of rheumatoid disease [2]. These patients should be monitored for up to two years after diagnosis of rice body formation as some of these patients have subsequently become symptomatic for RA [2]. Treatment for patients who are symptomatic involves surgical intervention, often leading to symptomatic relief [2].

## Conclusions

The salient point of this report is to highlight the importance of patient-specific differential diagnosis. While lipomas are a very common benign soft tissue tumor, patients with RA often have disease-specific sequelae that should be included in the diagnostic deliberation. While both lipoma and bursitis should be considered in the differential diagnosis for this patient, evaluation of a shoulder mass in a patient with RA is best assessed with MRI. Thus, when ordering diagnostic testing for patients with a palpable mass and rheumatoid arthritis, MRI--possibly preceded by conventional radiography--is the most appropriate diagnostic algorithm.
